# Spinal Hydatid Disease: A Case Report and Literature Review

**DOI:** 10.7759/cureus.94850

**Published:** 2025-10-18

**Authors:** Elmehdi Hamidi, Kenza Jamai, Yassine AIt M'barek, Lamia Benantar, Khalid Aniba

**Affiliations:** 1 Neurological Surgery, Ibn Tofail Hospital, Mohamed VIth University Hospital, Marrakech, MAR; 2 Neurological Surgery, Ibn Tofail Hospital, Mohammed VIth University Hospital, Marrakech, MAR

**Keywords:** albendazole, echinococcus granulosus, mri, parasitic infection, spinal cord compression, spinal hydatid disease, surgery

## Abstract

Spinal hydatid disease is a rare form of echinococcosis. This condition occurs when parasitic larvae migrate to the spinal column, leading to the formation of cysts that compress the spinal cord or nerve roots, resulting in neurological symptoms such as back pain, motor deficits, sensory changes, and sphincter dysfunction. A 45-year-old woman with a history of pulmonary and hepatic hydatid cysts, previously treated surgically, presented with progressive bilateral lumbosciatica and urinary incontinence. MRI revealed a hydatid cyst at the D1 vertebral level, prompting emergency surgery. The patient underwent D1-D2 laminectomy with excision of intact hydatid cysts, confirmed by histopathological examination. Postoperative treatment with albendazole resulted in significant clinical improvement within three months. Early diagnosis through imaging, particularly MRI, and prompt surgical intervention, combined with antiparasitic therapy, are critical for optimal recovery. This case underscores the importance of a multidisciplinary approach to the diagnosis and management of spinal hydatid disease, which remains a challenge in endemic areas but can have favorable outcomes with timely intervention.

## Introduction

Spinal hydatid disease, a rare manifestation of echinococcosis [1], is a parasitic infection caused by Echinococcus granulosus and Echinococcus multilocularis. While hydatid cysts most often affect the liver and lungs, their occurrence in the spine is extremely rare, representing less than 2% of all hydatid cases [2]. This condition occurs when the parasite larvae migrate to the spinal column, forming cysts that compress the spinal cord or nerve roots, leading to neurological symptoms such as back pain, motor or sensory deficits, and sphincteric dysfunctions. Diagnosis is mainly based on imaging examinations, particularly on MRI, and the treatment is usually a mix of surgical excision of cysts and anti-parasitic medication, for example, albendazole. The present case highlights the crucial role of an early diagnosis and quick intervention in order to prevent severe complications related to spinal cord compression and optimize the patient's recovery. The aim of this study is to illustrate the diagnostic and therapeutic challenges of spinal Echinococcosis, especially in endemic regions like Morocco, and to emphasize the importance of considering hydatid disease in the differential diagnosis of spinal lesions. By sharing this case, we seek to raise clinical awareness and outline key radiological and surgical features that support early management and help prevent irreversible neurological damage.

## Case presentation

A 45-year-old mother of two, who underwent surgery for pulmonary and hepatic hydatid cysts in June 2020, presented with bilateral lumbosciatica associated with sphincter disorders. Symptoms began six months ago in May 2024 with progressive bilateral lumbosciatica, rated 7 out of 10 on the visual analog scale (VAS), and associated with reduced walking distance and paresthesias of the lower limbs. Symptoms were marked by the progressive onset of sphincter dysfunction, particularly urinary incontinence. The neurological examination identified a dorsal spinal syndrome characterized by left-sided monoparesis, with segmental muscle forces: 4/5th, normal tone, regular walk without assistance, and urinary incontinence. The remainder of the clinical evaluation was unremarkable.

**Figure 1:**
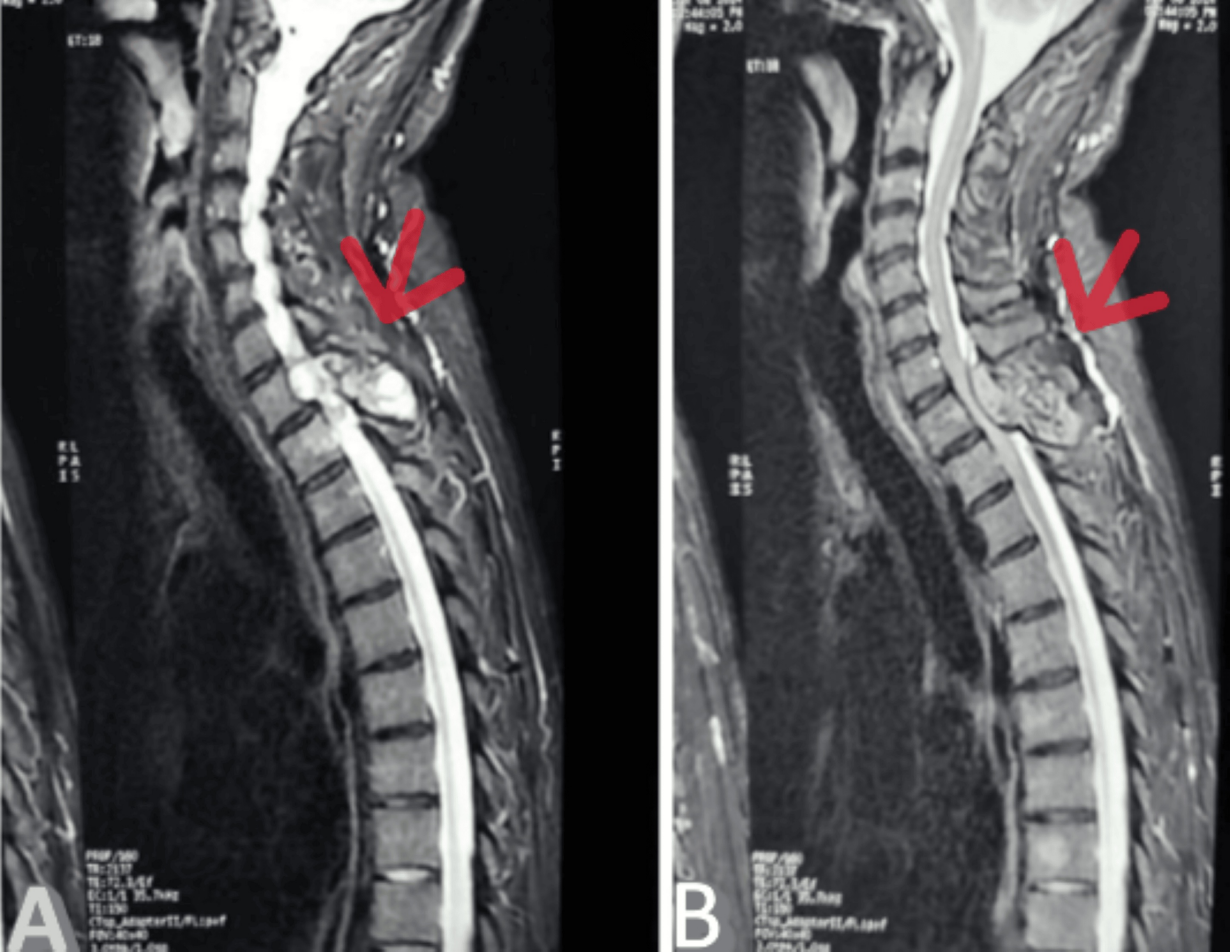
Cervicodorsal MRI in sagittal section showing a lesional process centered on the spinous process of D1 (A) Cervicodorsal MRI in sagittal section T2 sequence showing a lesional process centered on the spinous process of D1, which causes spinal cord compression. (B) Cervicodorsal MRI in sagittal section T1 sequence showing a lesional process centered on the spinous process of D1, which causes spinal cord compression.

Spinal magnetic resonance imaging (MRI) demonstrated a lesional process centered on the spinous process of the D1 vertebra, with bilateral intracanalar, resulting in compression of the spinal cord. This finding raised suspicion for a spinal hydatid cyst, particularly in light of the patient’s medical history of hydatid disease (Figure 1).

**Figure 2:**
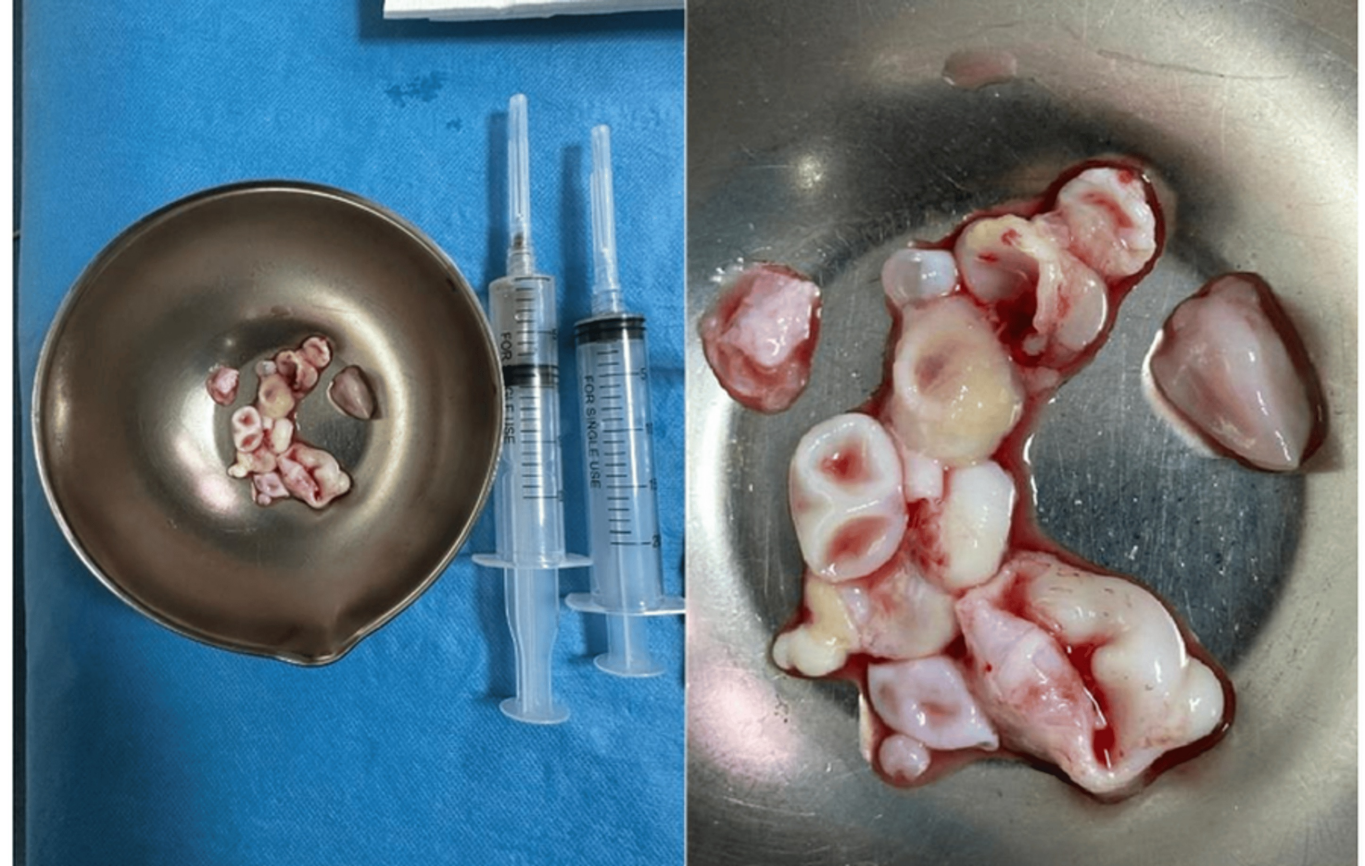
Image showing ruptured hydatid cysts after extraction The mass of hydatid cysts was extracted intact and then ruptured after extraction.

To assess the extent of the disease, a brain CT scan, chest X-ray, and abdominal ultrasound were performed. No abnormalities were identified in these tests. The hydatid serology was found to be positive. The patient underwent urgent surgery. The intervention involved a longitudinal incision from C7 to D2 with a D1-D2 laminectomy, which allowed for the excision of a mass of intact white cysts at the D1-D2 level, which ruptured after removal. The surgical site was thoroughly irrigated with hypertonic saline NaCl 20 to 30% and betadine solution (Figure 2).

Histopathological examination confirmed the presence of hydatid disease, with hydatid membranes observed within the excised cysts. The patient was initiated on medical treatment with the anti-helminthic agent: albendazole. Three months later, a marked clinical improvement was observed, with significant recovery of the neurological symptoms.

## Discussion

Spinal hydatid disease is a rare manifestation of echinococcosis, a parasitic infection caused by the Echinococcus species, primarily Echinococcus granulosus and Echinococcus multilocularis [2]. This disease occurs when the larvae of the parasite migrate to the spinal column and form cysts [1]. These cysts typically consist of a fluid-filled sac surrounded by a thick, fibrous capsule. In most cases, the infection affects the vertebrae and the epidural space, but it can also involve the intervertebral discs and the spinal cord [3]. The disease is often asymptomatic in its early stages, with clinical symptoms developing as the cysts grow and compress surrounding structures. Spinal hydatid disease is relatively rare, accounting for less than 2% of all hydatid cysts in humans [2]. It is more commonly observed in regions where Echinococcus is endemic (Africa, the Middle East, Central Asia, and South America) [4]. The infection is typically seen in rural areas with frequent livestock farming, as the parasite is transmitted through the ingestion of eggs shed by infected canines. The incidence of spinal hydatid disease is higher in regions with poor sanitation and close contact between humans, dogs, and livestock. The disease has a male predominance and typically affects individuals in the third to fifth decade of life [5].

The clinical presentation of spinal hydatid disease depends on the location and size of the cysts. Common symptoms include: (a) Back pain, often localized to the area of the affected vertebrae, (b) Neurological deficits, such as radiculopathy, paralysis, and sensory impairments, which occur if the cysts compress the spinal cord, (c) Motor dysfunction may result from direct pressure on the spinal cord, leading to weakness or even paraplegia in severe cases, (d) Progressive symptoms such as bowel or bladder incontinence may occur with the involvement of the conus medullaris or cauda equina [3], (e) Patients often present with a history of chronic back pain that worsens over time, along with signs of progressive neurological impairment [3].

The diagnosis of spinal hydatid disease primarily relies on imaging studies; MRI is the preferred diagnostic tool, as it offers superior soft tissue contrast and can visualize the cysts' characteristics, such as their fluid-filled nature, multilocular appearance, and membranes. The disease has a male predominance and typically affects individuals in the third to fifth decade of life [6]. On MRI, spinal hydatid cysts appear as well-defined lesions with a cystic structure that may cause compression of the spinal cord or nerve roots. CT scans can also be useful, especially for detecting bony involvement of the vertebrae, where osteolysis or destruction of the vertebral bodies may be visible [6]. In some cases, serologic tests such as ELISA can be used to support the diagnosis, but these tests may not always be reliable in patients with isolated spinal involvement [3].

The primary treatment for spinal hydatid disease includes antiparasitic medications, primarily albendazole, three courses at 800 mg/day for three months with a 15-day rest period [7]. Albendazole works by inhibiting the formation of the parasite's cell wall and is typically administered for several months, either as the sole treatment or as an adjunct to surgery. In some cases, praziquantel may also be used, though albendazole remains the drug of choice. These medications help reduce the cyst size and prevent the spread of infection [7].

Corticosteroids are often used to manage inflammation, particularly if cyst rupture occurs, which can lead to intense spinal cord edema and inflammation [8]. Surgical treatment is generally required for spinal hydatid disease, particularly in cases with large cysts causing compression of the spinal cord or nerve roots, or when the disease results in structural instability of the spine. Surgical goals include complete excision of the cysts while minimizing the risk of spillage of cyst contents, which can cause dissemination or the occurrence of anaphylactic shock that can be fatal for the patient [9]. Common surgical approaches include laminectomy or vertebrectomy, depending on the cyst’s location. Percutaneous aspiration may be an option for cysts that are more accessible or in cases where open surgery poses too high a risk. The use of hydatid fluid aspiration requires careful handling to avoid contamination of surrounding tissues [9].

Postoperative care for patients with spinal hydatid disease involves close monitoring for complications such as wound infection, spinal cord injury, or recurrence of the hydatid cysts. Regular follow-up with an MRI is essential to detect any recurrence or spread of the infection. After surgery, rehabilitation is crucial, especially if the patient has sustained significant neurological deficits, such as weakness or sensory loss [9].

The rehabilitation process may involve physical therapy to improve motor function and address any complications like muscle atrophy or paralysis. Long-term use of antiparasitic drugs may be required, particularly in patients who have had incomplete excision of the cysts or who develop new cysts during follow-up.

## Conclusions

Spinal hydatid disease, though rare, is a serious condition that demands early diagnosis and aggressive management. The clinical presentation often includes chronic back pain, neurological deficits, and progressive spinal cord compression, which can lead to permanent disability if untreated. Imaging techniques such as MRI play a crucial role in diagnosing spinal hydatid cysts, while surgical excision remains the cornerstone of treatment.

Antiparasitic medications, particularly albendazole, are used to reduce cyst size and prevent recurrence. In some cases, corticosteroids are employed to control inflammation, especially if the cysts rupture. Despite the challenges associated with this disease, outcomes can be favorable with timely intervention. However, recurrence remains a concern, and long-term follow-up is necessary to monitor for new cyst formation. This case emphasizes the importance of a multidisciplinary approach to the diagnosis and management of spinal hydatid cyst.

## References

[1] Kankam SB, Kheiri G, Safavi M, Habibi Z, Nejat F (2021). Isolated primary spinal epidural hydatid cyst in a child with progressive paraparesis. Childs Nerv Syst.

[2] Caglar YS, Ozgural O, Zaimoglu M, Kilinc C, Eroglu U, Dogan I, Kahilogullari G (2019). Spinal hydatid cyst disease: challenging surgery - an institutional experience. J Korean Neurosurg Soc.

[3] Pamir MN, Ozduman K, Elmaci I (2002). Spinal hydatid disease. Spinal Cord.

[4] Grosso G, Gruttadauria S, Biondi A, Marventano S, Mistretta A (2012). Worldwide epidemiology of liver hydatidosis including the Mediterranean area. World J Gastroenterol.

[5] Budke CM, Deplazes P, Torgerson PR (2006). Global socioeconomic impact of cystic echinococcosis. Emerg Infect Dis.

[6] Teke M, Göçmez C, Hamidi C (2015). Imaging features of cerebral and spinal cystic echinococcosis. Radiol Med.

[7] Velasco-Tirado V, Alonso-Sardón M, Lopez-Bernus A (2018). Medical treatment of cystic echinococcosis: systematic review and meta-analysis. BMC Infect Dis.

[8] Sengul G, Kadioglu HH, Kayaoglu CR, Aktas S, Akar A, Aydin IH (2008). Treatment of spinal hydatid disease: a single center experience. J Clin Neurosci.

[9] Kalkan E, Keskin F, Erdi F (2014). Surgical treatment of spinal hydatidosis. Turgut M.

